# Exploiting the potential of lung stem cells to develop pro-regenerative therapies

**DOI:** 10.1242/bio.059423

**Published:** 2022-10-14

**Authors:** Robert E. Hynds

**Affiliations:** ^1^ Epithelial Cell Biology in ENT Research (EpiCENTR) Group, Developmental Biology and Cancer Department, UCL Great Ormond Street Institute of Child Health, University College London, London, WC1N 1DZ, UK; ^2^ UCL Cancer Institute, University College London, London, WC1E 6DD, UK

**Keywords:** Epithelial stem cells, Lung regeneration, Regenerative medicine, Respiratory biology, Cell transplantation

## Abstract

Acute and chronic lung diseases are a leading cause of morbidity and mortality globally. Unfortunately, these diseases are increasing in frequency and we have limited treatment options for severe lung diseases. New therapies are needed that not only treat symptoms or slow disease progression, but also enable the regeneration of functional lung tissue. Both airways and alveoli contain populations of epithelial stem cells with the potential to self-renew and produce differentiated progeny. Understanding the mechanisms that determine the behaviour of these cells, and their interactions with their niches, will allow future generations of respiratory therapies that protect the lungs from disease onset, promote regeneration from endogenous stem cells or enable regeneration through the delivery of exogenous cells. This review summarises progress towards each of these goals, highlighting the challenges and opportunities of developing pro-regenerative (bio)pharmaceutical, gene and cell therapies for respiratory diseases.

## Teaser

**Summary:** Lung regeneration by tissue-resident stem cells represents a therapeutic target to reverse the functional decline of lung tissue in respiratory diseases. This review covers approaches to improve regenerative outcomes by promoting the function of endogenous cells, creating a regeneration-competent microenvironment or applying exogenous stem cells in cell therapy approaches.

## Introduction

Approximately 7.4% of the human population are currently affected by a chronic lung disease, making respiratory disease a major cause of morbidity and mortality globally (GBD Chronic Respiratory Disease Collaborators, 2020). Pathologies affecting the large airways, small airways and/or alveoli can all lead to a reduced volume of functional lung tissue. This impairs the vital functions of gas exchange, mucociliary clearance, and immune cell production. The proximal airways can be affected by genetic diseases, such as cystic fibrosis (CF) and primary ciliary dyskinesia, and are the site of origin for squamous cell lung cancer. Small airways develop pathology in chronic obstructive pulmonary disease (COPD) and bronchiolitis obliterans, while alveolar injury is implicated in acute respiratory disease (ARDS), COPD, pneumonia and interstitial lung diseases.

Current therapies for progressive lung diseases target inflammation, bronchoconstriction or fibrosis, with a view to slowing disease progression and/or controlling symptoms. During end-stage lung disease, heart–lung transplantation can provide an extreme option to provide patients with healthy lung tissue but donor shortages, immunosuppression and the risk of rejection remain significant limitations of this approach. Since no therapy has yet proved capable of reversing established disease, the generation of ‘pro-regenerative’ therapies – those that actively promote wound healing and tissue repair to increase the volume of functional tissue – is a major aim for the field. A pro-regenerative effect could be achieved by modulating lung stem cell function, for example by protecting or stimulating endogenous stem cells, or by engrafting exogenous stem cells. Such a therapy could provide an entirely novel approach or act as an adjunct to existing therapies.

Over the last two decades, significant progress has been made in understanding the cellular and molecular basis of lung regeneration through a combination of improved *in vitro* modelling using human cell models, *in vivo* animal models, and technological advances. In light of this recent progress, this article reviews existing and potential approaches to modulating epithelial regeneration in the lung as a route to preventing or treating respiratory diseases.

## Lung stem cells

**Fig. 1.**
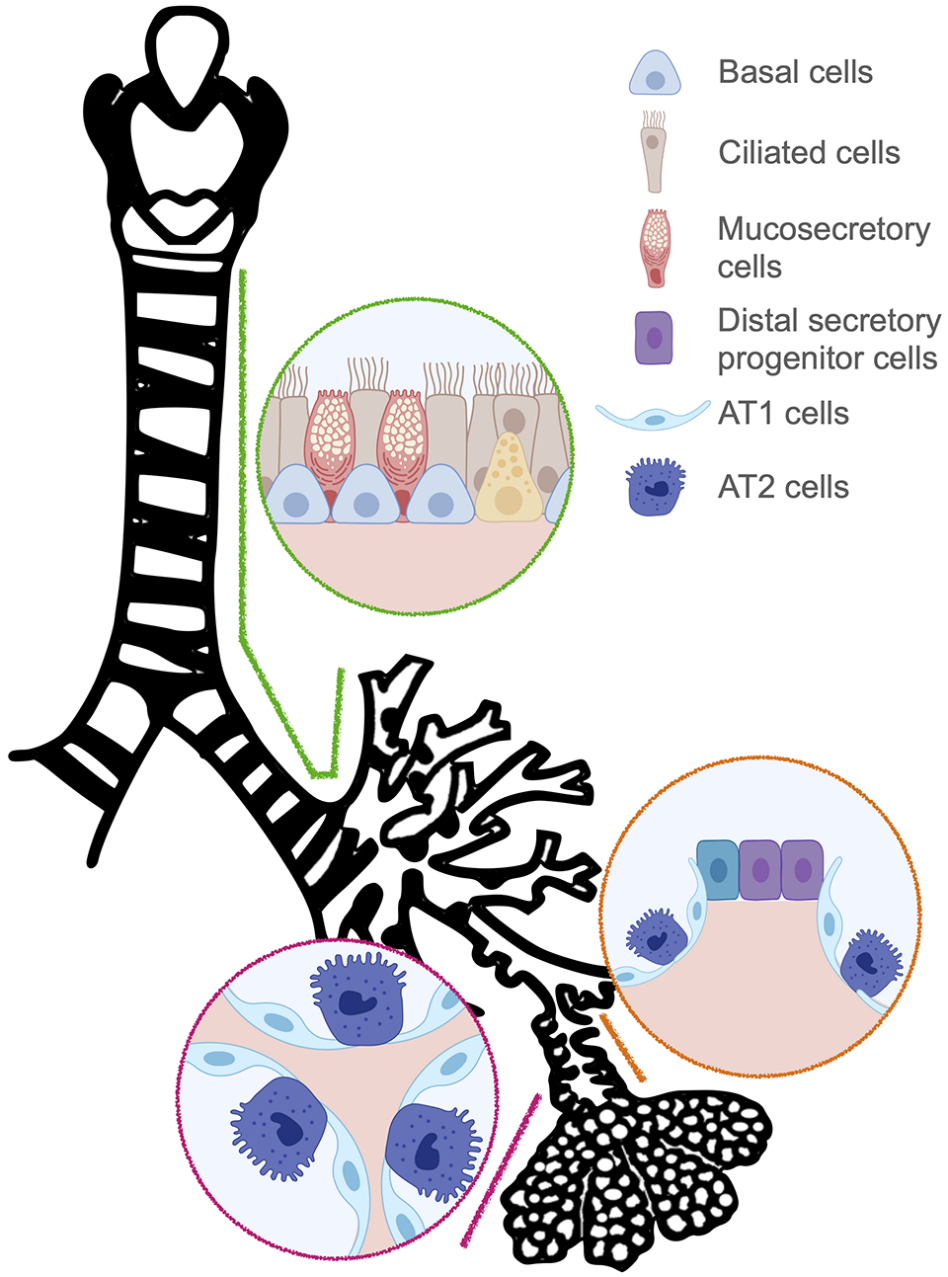
**Stem cells within the respiratory tree.** Large airways (green), small airways (orange) and alveoli (magenta) harbour independent populations of stem cells during homeostasis. Following severe lung injury, established stem cell hierarchies adapt flexibly with cells from the airways capable of contributing to alveolar repair.

The epithelia of the nose, larynx, trachea, bronchi and alveolus are important interfaces with the outside world. These barriers are charged with host defence against inhaled pathogens and particulates, as well as enabling pulmonary gas exchange. During homeostasis, the turnover of the airway and lung epithelia is relatively low compared to skin or gut epithelia, but region-specific stem cell populations maintain lung epithelia and repair them following injury (Fig. 1) (Basil et al., 2020).

Human proximal airways are lined by a pseudostratified epithelium that is maintained by TP63-expressing basal stem cells (Rock et al., 2009). Comparable cellular composition and stem cell behaviour is observed in the mouse trachea, although there are significant species differences in respiratory stem cell biology (reviewed in Basil and Morrisey, 2020).

At airway termini, respiratory bronchioles containing simple columnar epithelium bridge the airway and alveolar compartments. Human respiratory bronchioles contain a population of secretory progenitor cells with the capability to generate alveolar type II (AT2) in organoid models (Basil et al., 2022). The plasticity of this region is underscored by the finding that AT2 cells can give rise to secretory cells found within respiratory bronchioles (Kadur Lakshminarasimha Murthy et al., 2022). However, the relationship of these populations with each other and the extent to which they are analogous to bronchioalveolar stem cells identified in mice (Kim et al., 2005), remain to be determined.

In the alveolus, the epithelium consists of alveolar type I (AT1) and AT2 cells, which are specified during embryonic development and continue to mature following birth. AT1 cells are large, thin cells that provide the majority of the alveolar surface area at the interface between air and pulmonary capillaries. AT2 cells are comparatively small, cuboidal in shape and are responsible for the synthesis and secretion of pulmonary surfactant. AT2 cells can proliferate during postnatal life, giving rise to AT1 and AT2 cell populations (Barkauskas et al., 2013). While AT1 cells have traditionally been viewed as a terminally differentiated population, they can be mobilised for regeneration in specific circumstances, including hyperoxia (Penkala et al., 2021) and during post-pneumonectomy compensatory lung growth (Jain et al., 2015; Wang et al., 2018).

## Promoting lung regeneration by endogenous stem cells

### Effects of existing respiratory therapies on lung regeneration

In COPD, chronic small airway inflammation leads to airway wall thickening and fibrosis (Barnes et al., 2015). Progression involves the loss of respiratory bronchioles and alveolar destruction (emphysema), which results in loss of lung function. Current therapies for COPD aim to prevent declining lung function, control symptoms and reduce the frequency of exacerbations. Therapies include corticosteroids to suppress inflammation, and anti-cholinergic and β2-agonist bronchodilators to induce airway smooth muscle relaxation (Barnes et al., 2015).

While these therapies do not target epithelial stem cell populations directly, all are likely to impact epithelial homeostasis and/or repair. For example, while corticosteroids are used to curb inflammation in COPD, they are also used foetal lung maturation in women at risk of preterm delivery (McGoldrick et al., 2020). In the latter setting, corticosteroids induce the expression of surfactant proteins, lung beta adrenergic receptors and genes associated with phospholipid synthesis. The mechanism remains unclear but effects of corticosteroids on stem cell emergence, proliferation and differentiation are supported by the findings that dexamethasone-containing maturation cocktails induce AT2 cells in primary foetal cell culture (Gonzales et al., 2002) and induce alveolar differentiation from NKX2.1+ progenitors during step-wise human induced pluripotent cell (iPSC) differentiation (Schmeckebier et al., 2013). How corticosteroid treatment might influence stem cell fate in adult COPD lungs remains unclear but further study of the effects of corticosteroids on lung stem cell behaviour may be informative.

Bronchodilators are used to decrease resistance in the lung and improve airflow. Broadly, these drugs either inhibit cholinergic or activate β2-adrenoreceptors (β-ARs) to induce the relaxation of airway smooth muscle. The muscarinic receptors targeted by anti-cholinergic bronchodilators are also expressed on airway epithelial cells, including ciliated cells where they regulate ciliary beat frequency. Though expressed by basal stem cells, any role muscarinic receptors have in stem cell function is so far uncharacterised. β2-agonist bronchodilators – which are the most heavily prescribed bronchodilators for asthma – activate receptors on airway smooth muscle cells to relax airways. However, activation of the same receptors on epithelial cells contributes to the inflammation, mucous metaplasia and airway hyperresponsiveness associated with asthma (Nguyen et al., 2017). Moreover, investigation of their effects on airway basal cells revealed that β-AR agonists reduced migration and wound closure in airway epithelial cells (Peitzman et al., 2015), suggesting that these therapies might negatively influence epithelial regeneration in COPD and asthma. Mechanistic experiments revealed that combining β-AR agonists with a PP2A inhibitor blocked the wound closure delay, suggesting that novel β-AR agonists that induce cAMP accumulation but do not stimulate PP2A activity might counter bronchoconstriction without negatively impacting epithelial regeneration (Peitzman et al., 2015). β-adrenergic receptors are expressed at even higher levels in the distal lung epithelia. While little is known about any effect(s) that they may have on small airway or alveolar stem cell function, it would be possible to investigate these issues in precision-cut lung slices (Alsafadi et al., 2020).

In 2014, the small molecules pirfenidone and nintedinab were approved as anti-fibrotic therapies for pulmonary fibrosis. Pirfenidone was identified as an anti-inflammatory compound and was later shown to be anti-fibrotic in animal models (Schaefer et al., 2011). While its molecular target is not known, the drug reduces fibroblast proliferation and myofibroblast differentiation (Conte et al., 2014). Nintedinab, on the other hand, is a broad, competitive inhibitor of tyrosine kinases [including vascular endothelial growth factor receptors (VEGFRs), fibroblast growth factor receptors (FGFRs) and platelet derived growth factor receptors (PDGFRs)], making an effect on epithelial stem cells in the fibrotic lung likely. Consistent with this, nintedanib but not pirfenidone increased the abundance of pro-surfactant protein C (SP-C) and the expression of AT2 cell markers in precision-cut lung slices from bleomycin-treated mice or human lung tissue that had been treated with pro-fibrotic factors (Lehmann et al., 2018). Whether nintedanib increased AT2 marker gene expression on a per cell basis or led to increased AT2 cell abundance is not known, but these data demonstrate the relevance of studying the effects of putative therapies on lung stem cell function during the drug development process.

Overall, the effects of clinical therapies on lung regeneration are currently only partially understood and furthering this understanding might enable combination therapies that reduce inflammation and/or fibrosis as well as potentiate epithelial repair.

### Developing new pharmacologic therapies to promote lung regeneration

Ultimately, progress in the treatment of established chronic lung diseases will require therapies that actively promote lung repair and regeneration to reverse tissue damage. The ability to modulate stem cell proliferation, migration and/or differentiation would be beneficial to this aim. We increasingly understand the signals that control lung stem cell behaviour and these mechanisms often mirror those used during lung development (Zepp and Morrisey, 2019).

A range of cell types contribute to a complex microenvironment in which lung regeneration occurs; lung epithelial stem cell niches consist of differentiated epithelial cells (Pardo-Saganta et al., 2015), mesenchymal (Lee et al., 2017) and endothelial cells (Lee et al., 2014; Cao et al., 2016), airway smooth muscle (Volckaert et al., 2011), T regulatory cells (Mock et al., 2014) and macrophages (Engler et al., 2020; Lucas et al., 2022). Within this environment, proliferation of lung stem cells is promoted by epidermal growth factor (EGF) (Brechbuhl et al., 2014; Ding et al., 2011; Vermeer et al., 2003), FGF (Balasooriya et al., 2017; Ulich et al., 1994), hepatocyte growth factor (HGF) (Ohmichi et al., 1996), BMP (Chung et al., 2018; Mou et al., 2016) and Wnt signalling (Aros et al., 2020; Jensen-Cody et al., 2021; Nabhan et al., 2018; Zacharias et al., 2018), while mechanisms such as FGFR1 (Balasooriya et al., 2016) and p53 (McConnell et al., 2016) signalling also act to restrain stem cell proliferation. Acute TGFβ drives a pro-migratory phenotype in airway basal cells, contributing to wound repair (Pittet et al., 2001) but chronic TGFβ signalling activation can also inhibit epithelial regeneration (Riemondy et al., 2019). Notch signalling (Rock et al., 2011) and the cytokine milieu influence cell fate; interleukin 6 (IL-6) and IL-13 have opposing effects on airway differentiation trajectory, promoting ciliogenesis (Tadokoro et al., 2014) and mucosecretory fate (Atherton et al., 2003), respectively.

Several of these pathways have been investigated as potential targets in pre-clinical chronic lung disease studies. Most notably, evidence that all-trans retinoic acid (ATRA) stimulated alveolar repair in an elastase-induced emphysema model in rats (Massaro and Massaro, 1997) led to clinical studies in COPD that aimed to promote lung repair using either ATRA (Mao et al., 2002; Roth et al., 2006) or palovarotene (Stolk et al., 2012), a RAR-γ agonist. Unfortunately, these interventions did not achieve impact on either lung volume or function measures, although the drugs were well tolerated and some suggestions of slowed disease progression were noted. Subsequent studies showed that effects in animal models were highly strain dependent (Stinchcombe and Maden, 2008), and *in vitro* studies suggested that RA pathway activation in fact decreased lung epithelial organoid size while pathway inhibition could increase epithelial cell proliferation (Ng-Blichfeldt et al., 2018). Moreover, combining RA pathway inhibition with histone deacetylase (HDAC) inhibition restored the differentiation capacity of larger organoids (Ng-Blichfeldt et al., 2018), suggesting that a more nuanced, sequential approach to RA pathway manipulation might be more beneficial.

The example of ATRA speaks to a wider challenge in developing a pro-regenerative therapy, rather than one that alleviates symptoms or slows disease progression: a single intervention can have conflicting effects on different lung cell types. This is exemplified by work that identifies the EGFR signalling pathway as a potential target in chronic lung disease. Inducible lung epithelial expression of TGF-α can drive lung fibrosis (Hardie et al., 2004), while small molecule inhibition of MEK (Madala et al., 2012) or PI3K (Le Cras et al., 2010) can delay disease progression. However, these pathways are vital for epithelial stem cell proliferation (Brechbuhl et al., 2014), which might in part explain why cessation of TGF-α overexpression reverses fibrosis whereas small molecule therapies only delay progression (Madala et al., 2012). As such, identifying targets without significant off-target effects on regenerative potential, or targeting therapies to specific cell subsets could be valuable.

Lung epithelial regeneration has been improved in animal studies using both biopharmaceuticals and small molecule approaches. Biopharmaceutical approaches shown to increase epithelial cell proliferation after lung injury have included keratinocyte growth factor (KGF, Kaza et al., 2002; HGF, Dohi et al., 2000) and vascular endothelial growth factor (VEGF, Kunig et al., 2005). The small molecule LDN-193189 – a BMP antagonist – promoted basal cell proliferation during airway injury (Tadokoro et al., 2016), while amlexanox – a Wnt-activator – was identified in a drug repurposing study and promoted repair in the elastase-induced emphysema mouse model (Costa et al., 2021). In combination, it is possible to imagine a future therapeutic approach which combines complementary anti-inflammatory/anti-fibrotic therapy with a second approach to promote regeneration. However, care will be required in the design of such an approach given that persistent epithelial repair can be a driver of disease. Rather than proliferation, such a therapy might also target epithelial cell differentiation. A recent investigation showed that targeting epithelial cell proteostasis with a small molecule inhibitor of the integrated stress response (ISRIB) that relieves inhibition of translation can promote AT2-to-AT1 differentiation, which in turn inhibits macrophage- and fibroblast-driven fibrotic responses (Watanabe et al., 2021). This demonstrates the promise of approaches that target epithelial regeneration to promote disease resolution.

Several other aspects of lung epithelial biology might offer new approaches to promoting lung regeneration. First, the remarkable plasticity of cells following injury may enable the reprogramming of differentiated epithelial cells to a stem cell state. Cells committed to a secretory fate are able to regenerate mouse airways following extreme damage (Tata et al., 2013), AT1 cells able to generate AT2-like cells following loss of YAP/TAZ (Penkala et al., 2021) and high cellular plasticity is observed at the transition zone between airways and alveoli (Basil et al., 2022; Kadur Lakshminarasimha Murthy et al., 2022), overturning the decades-old paradigm of unidirectional stem cell differentiation. Understanding and ultimately modulating this plasticity might become an important tool to broaden the range of cell types that act as targets for pro-regenerative therapies. Second, appreciation of stem cell heterogeneity and its role in disease states offer a potential new avenue for exploration. Recent data demonstrate that clonally derived basal stem cells from COPD patients belong to one of at least four subtypes with distinct transcriptomic and functional phenotypes that are maintained in cell culture and after transplantation in mouse models (Rao et al., 2020). In addition to seemingly healthy cells, COPD patient lungs contained variant clones with a propensity for goblet cell hyperplasia, squamous cell metaplasia or squamous cell metaplasia with inflammation (Rao et al., 2020). The co-existence of stem cells without pathogenic features with these variant subtypes in patients raises the possibility of developing therapies that target aberrant basal cells but spare healthy stem cells (Rao et al., 2020). Finally, disease heterogeneity might be exploited. The distribution of lesions in the lungs of pulmonary fibrosis patients is spatially heterogenous, with emphysema presenting in the upper lobes and fibrosis occurring in the lower lobes. Since disease pathogenesis is thought to involve impaired epithelial stem cell function as a result of repetitive injury, the relative sparing of some spatial regions raises the possibility that stem cells within these regions might be mobilised to repair damaged tissue or to repopulate lung regions following ablative therapy. However the extent of lung cell motility and migration *in vivo* are not known and our understanding of the mechanisms that control cell movement is limited.

## Modulating the lung stem cell niche to create a regeneration-competent microenvironment

So far, this review has considered approaches to encourage regeneration by resident stem cells directly. However, modulating the lung microenvironment or protecting stem cells from local inflammation might also prove effective in improving regenerative outcomes and/or avoiding the initiation of damaging feed-forward cycles that drive disease progression.

Skeletal stem cells (SSCs) – or ‘mesenchymal stromal cells’ (MSCs) – isolated from the bone marrow have been investigated as cell therapies in a wide range of settings due to their ease of isolation and expansion (De Luca et al., 2019). In lung disease models, including the elastase-induced lung injury and the inflammatory phase of bleomycin-induced injury, intravenous injection of SSCs can improve functional measures, improve survival and/or reduce inflammation (reviewed in Geiger et al., 2017). The widely held view is that cell therapy acts to modulate the regenerative response via paracrine effects mediated by growth factor, cytokine and extracellular vesicle production. Although SSC therapy has proven safe in clinical studies, disease-modifying effects in pre-clinical models have failed to be translated to human clinical trials. Although SSCs may yet be modified for therapeutic effect in humans (Rolandsson Enes et al., 2021), it should also be possible to study, and eventually target, specific pathways by which they improve lung regeneration in pre-clinical models.

During homeostasis and wound healing, growth factors produced by fibroblasts are key niche factors, but their expression is abnormally low in diseased lung tissue (Marchand-Adam et al., 2003). Knockdown of HGF prior to SSCs therapy reduces their protective effect in both the elastase-induced lung injury model (Kennelly et al., 2016) and the bleomycin lung injury model (Cahill et al., 2016), identifying the supplementation of growth factors from the healthy stem cell niche as a possible avenue for therapy. Indeed, application of HGF in the lung bleomycin injury model protects mice from fibrosis (Dohi et al., 2000). Like HGF, KGF is an epithelial mitogen that is predominantly produced by lung fibroblasts. Pre-administration of KGF protects against hyperoxia (Panos et al., 1995), acid instillation (Yano et al., 1996), radiation (Yi et al., 1996) and bleomycin-induced lung injury (Yi et al., 1996). The protective effect of KGF appear to be multi-faceted, with roles for stimulating proliferation of AT2 cells, inhibiting apoptosis, reducing oxidative stress and limiting DNA damage (Finch and Rubin, 2004). Further, KGF pre-treatment improved bacterial clearance and alveolar macrophage function in response to Escherichia coli infection (Gardner et al., 2016). Encouragingly, the increased alveolar proliferation and production of immune mediators seen in animal models were also observed in a lipopolysaccharide (LPS) human-challenge model in which KGF was administered prior to LPS exposure (Shyamsundar et al., 2014). However, the protective effect of KGF is not universal across different types of challenge, as KGF administration actually increases susceptibility to influenza infection (Nikolaidis et al., 2017). Thus suppressing AT2 proliferation might be protective during viral infection. Consistent with this, the authors note that targeting PI3K/Akt signalling with rapamycin has proven beneficial in severe influenza-driven pneumonia (Nikolaidis et al., 2017; Wang et al., 2014). While pre-application of the niche factors HGF and KGF appears to prime lung stem cells for successful regenerative outcomes, this approach has seen limited clinical applicability due to the nature of real-world lung injuries.

Since chronic inflammation is a feature of the lung stem cell niche in both ageing and chronic lung disease, this also represents a target to improve the regenerative microenvironment. For example, the inflammatory cytokine interleukin 1 beta (IL-1β) is elevated in bronchoalveolar lavage fluid from healthy smokers (Kuschner et al., 1996) and IPF patients (Wilson et al., 2010), as well as tissue from COPD patients (Pauwels et al., 2011), suggesting inadequate resolution of inflammation. Notably, IL-1β primes AT2 cells for differentiation, but chronic exposure to IL-1β inhibits full differentiation to AT1 cells and halts cells in a progenitor state in which they might be vulnerable to DNA damage (Choi et al., 2020). As such, the suppression of chronic inflammation might allow resolution of epithelial cell differentiation and reduce disease risk. Evidence to support this was found in the CANTOS trial, which assessed whether canakinumab (an anti-IL-1β monoclonal antibody) prevented recurrent vascular events in patients who had suffered from myocardial infarctions (Ridker et al., 2017a). Patients treated with canakinumab had a lower risk of lung cancer incidence and mortality than those in the placebo group (Ridker et al., 2017b), suggesting that IL-1β might promote lung cancer initiation and/or progression. Further studies are required to interrogate how immune-epithelial stem cell interactions are altered during chronic inflammation and how these might be targeted to promote regeneration.

A further major alteration to the stem cell niche that occurs during both ageing and chronic lung diseases is the onset of cellular senescence. Senescence-associated phenotypic changes, such as cell cycle arrest and senescence-associated secretory phenotypes, are likely to have evolved at least in part to limit regeneration from damaged cells by encouraging their clearance by the immune system, thus promoting productive tissue repair following acute injury (Rodier and Campisi, 2011). However, just as persistent IL1β stimulation becomes problematic, persistent senescence during ageing or chronic injury, can drive pathology and reduce regenerative capacity. The epithelial cells overlying the hallmark fibrotic lesions in pulmonary fibrosis express markers of senescence and, in fibrosis models, induction of senescence in AT2 cells drives fibrosis (Yao et al., 2021). Likewise, increased numbers of both airway epithelial (Birch et al., 2015) and AT2 (Tsuji et al., 2006) cells have senescent phenotypes in patients with COPD. Promisingly, removal of senescent cells from the lungs using either genetic ablation or treatment with senolytic drugs, such as dasatinib and quercetin, can restore stem cell markers and improve lung function in experimental models of both pulmonary fibrosis (Lehmann et al., 2017; Schafer et al., 2017) and COPD (Ganesan et al., 2010), and clinical trials are ongoing (Justice et al., 2019). Since senescence is also a hallmark of pre-invasive squamous cell lung cancer lesions (Venkatesan et al., 2021) and senolytic CAR-T cells have shown promise in pre-clinical models (Amor et al., 2020), senolytic therapy may also provide an opportunity to intercept lung cancers at an early stage of development, avoiding invasive bronchoscopic or even surgical procedures (Thakrar et al., 2017). A logical extension of these studies in established disease will be the investigation of senolytic drugs as prophylactic therapies. Since ageing leads to the accumulation of senescent cells and their removal improves disease outcomes, could intervention in high-risk groups reduce the lung disease burden?

Genomic analysis of human airway basal cells revealed that tobacco smoking leads to increased genomic damage with a high degree of cell-to-cell variability such that some basal cells remain relatively spared while others carry thousands of mutations (Yoshida et al., 2020). Importantly, basal cells with less genomic damage were more prevalent in the airways of ex-smokers than current smokers (Yoshida et al., 2020), suggesting that removal from the smoking-associated microenvironment might favour their expansion at the expense of more heavily damaged cells. These findings raise multiple potential questions and opportunities relevant to lung regeneration. First, are cells with a high mutational load senescent and could targeting these cells to enable regeneration by those with less damage reduce the risk of cancer? Second, how are some cells protected from genomic damage during the chronic injury state induced by smoking but not others? Determining this might enable approaches to protect lung stem cells from damage in a wider range of settings, including following exposure to air pollution or occupational hazards, and during chemo/radiotherapy. However, further research is required to distinguish between multiple possibilities, including that these cells are physically protected by their niche (e.g. in submucosal glands), mitotically quiescent or of a subtype of basal cells that is defined by intrinsic protection.

## Promoting lung regeneration using exogenous stem cells

Following lung damage in which resident stem cells have been depleted or are dysfunctional, lung regeneration might also be stimulated by transplanting epithelial stem cells into the lung. Potential rationales for cell therapy include repopulation of lung epithelia following extensive injury, the suppression of inflammation and/or fibrosis, and to treat genetic disorders. Indeed, combined cell and gene therapy approaches in which cells are gene-corrected *ex vivo* might overcome some of the limitations of gene therapy, such as the physical barrier to transduction posed by airway mucous and luminal epithelial cells (King et al., 2020).

Stem cell therapy using exogenous cells, in the form of bone marrow or haematopoietic stem cell (HSC) transplantation, is an approved therapy in the context of haematopoietic malignancies, while epithelial stem cells have been successfully employed for severe burns injuries and limbal stem cell insufficiency (Hynds et al., 2018). For a small number of patients with epidermolysis bullosa who have mutations in the gene encoding the laminin-332 component LAMB3, it has been possible to replace either small (Mavilio et al., 2006) or substantial (Hirsch et al., 2017; Kueckelhaus et al., 2021) proportions of the patients’ skin with cultured, retrovirally transduced epidermal cells expressing the wildtype LAMB3 protein. While these studies show that epithelial cell transplantation can be feasible, safe and efficacious, multiple challenges remain to using epithelial cells clinically in the context of the respiratory system.

Firstly, there are multiple possible cell types that might be used for transplantation. Until recent protocol breakthroughs, human primary airway basal cells have had limited culture spans and protocols for the long-term expansion of human alveolar epithelium had not been developed. This led to the consideration of iPSCs as a potential source, since both airway basal cells (Hawkins et al., 2021) and AT2 cells (Jacob et al., 2017) can be derived from iPSCs. However, issues around cell number, purity and plasticity, as well as safety concerns, limit their translational potential (reviewed in Berical et al., 2019). When considering primary cells, the lack of culture conditions proven to expand stem cells capable of long-term regeneration *in vivo* poses a further challenge. Learning from other epithelial tissues, we now appreciate that maintaining stem cells with long-term repopulating capacity through culture steps determines graft outcome (Pellegrini et al., 2001). Transplantation of differentiated airway epithelium after air-liquid interface culture showed only short-term survivability in animal models (Hamilton et al., 2020), suggesting that isolation, expansion and transplantation of long-term self-renewing basal cells with differentiation occurring post-transplantation is preferable for therapeutic applications (Hayes et al., 2019; Ghosh et al., 2017). The application of basal cells in airway regenerative approaches is also supported by the availability of well-established protocols, including some similar to methods used clinically for skin (Rao et al., 2020; Butler et al., 2016), but further work is required to determine robust criteria that define stem cells suitable for transplantation in terms of their long-term repopulating capacity *in vivo*.

While alveolar epithelial cells can be expanded long-term in organoid cultures (Salahudeen et al., 2020; Katsura et al., 2020; Youk et al., 2020), the scalability of this approach to clinically relevant cell numbers and demonstration of their potential to maintain long-term tissue renewal *in vivo* has not been proven. Promisingly, mouse AT2 cells can be cultured as organoids, transplanted in the bleomycin lung injury model and both contribute to alveologenesis following further injury and retain their capacity to form organoids *in vitro* (Louie et al., 2022). Consistent with the above-mentioned failure to engraft differentiated airway cells, AT1 cells from organoid cultures do not engraft in the lung bleomycin model (Louie et al., 2022), suggesting that maintaining AT2 cells with long-term repopulating capacity will be essential during protocol development and will form an important aspect of quality control processes. As yet, data for adult human organoid transplantation are lacking and compatibility of these protocols with GMP-manufacture standards has yet to be demonstrated, posing an additional challenge to their translation to clinical practice.

Secondly, stem cell transplantation protocols to date have required pre-conditioning of the recipient. Likewise, in the lung, it has not proven possible to transplant cells without pre-conditioning by causing epithelial damage, for example using polidocanol, naphthalene, bleomycin or influenza (Table 1) to target different lung epithelia. The site of injury determines the niche available for transplanted cells and therefore the location of engraftment. However, the use of these poses a challenge for clinical transplantation, as these methods are not likely to be suitable as clinical pre-conditioning regimens. Promisingly, there is precedent for deploying autologous epithelial cell grafts during airway surgery, where split-skin grafts have been used as ‘biological dressings’ to inhibit inflammation and fibrosis in laryngotracheal stenosis in both paediatric (Bowe et al., 2018) and adult (Davis et al., 2022; Nouraei and Sandhu, 2013) populations. During these procedures, airways were pre-conditioned using a microdebrider to disrupt the epithelial surface, which provides a starting point for pre-clinical research to optimise airway pre-conditioning.

**Table 1.**
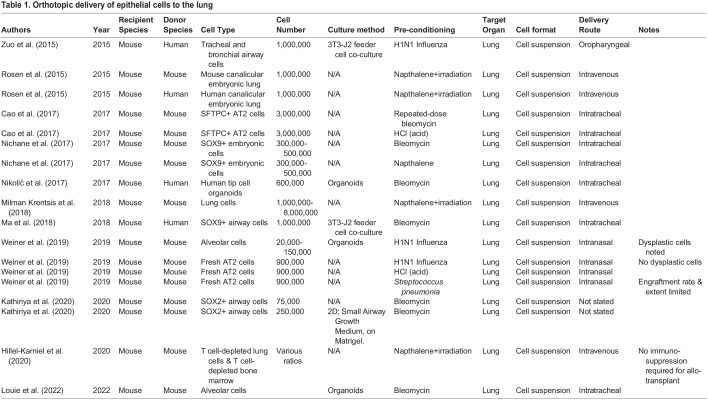
Orthotopic delivery of epithelial cells to the lung

Thirdly, since the lungs have evolved mechanisms to protect us against exogenous material, cell delivery poses an additional challenge to both airway and lung cell transplantation protocols. In target patient populations, this challenge is exacerbated by the unreceptive microenvironment presented by inflammation, excess mucous, and/or infection. Notably, the fate of transplanted epithelial cells in bioengineered large airways (Hamilton et al., 2015) and in two bronchiectasis patients who received autologous airway basal cells via fibreoptic bronchoscopy (Ma et al., 2018) is unknown with little evidence to suggest engraftment, although clinical trials involving similar methods are ongoing (NCT02745184, NCT03153800). Modelling cell transplantation in a clinically translatable manner is challenging, with a majority of studies deploying single-cell suspensions delivered via the trachea (Table 1). On the other hand, the use of cell-seeded scaffolds or sheets, such as fibrin (Ronfard et al., 2000) or porcine small intestinal submucosal membrane (Vaidyanathan et al. 2020), might be clinically applicable to airways, but neither approach has yet to be tested *in vivo* for lung regeneration. In many scenarios in which epithelial cell transplantation to airways would be desirable, stenting is required to maintain a patent airway. The stents combined with movement generated by breathing and coughing likely cause substantial friction forces over the epithelial surface.

A final hurdle to consider in the design of lung epithelial cell therapies is their interaction with endogenous epithelial cells post-transplantation. In epidermolysis bullosa, cell therapy has corrected mutations associated with a clear phenotypic disadvantage, a failure of adhesion to the basement membrane due to an absence of laminin-332. This gives transplanted cells a plausible competitive fitness advantage over the mutant host cells. However, combined cell and gene therapy using AAVs, lentiviruses or CRISPR-based approaches has also been extensively investigated in the setting of cystic fibrosis, a disease that is caused by mutations in the cystic fibrosis transmembrane conductance regulator (CFTR) gene (reviewed in Berical et al., 2019). Here, the fitness advantage conferred by gene correction is not obvious given that CFTR channels are predominantly expressed in differentiated epithelial cell types. Indeed, the cell culture process might produce a competitive fitness disadvantage. In the future, understanding the mechanisms of cell competition in adult airway epithelium might enable the selection of clones for transplantation based on their fitness or the manipulation of transplanted cells to induce ‘super-competition’ over non-transplanted host cells.

Overall, substantial progress has been made towards cell-based therapy in the lung over the past decade and focussed pre-clinical work on pre-conditioning, model development and cell delivery are likely to lead to translational breakthroughs. Substantial progress is also being made in the field of lung bioengineering (reviewed in De Santis et al., 2018), such that ultimately it might be possible to combine epithelial stem cell transplantation with biomaterials and additional cell types in order to generate new functional lung tissue.

## Conclusion

**Fig. 2.**
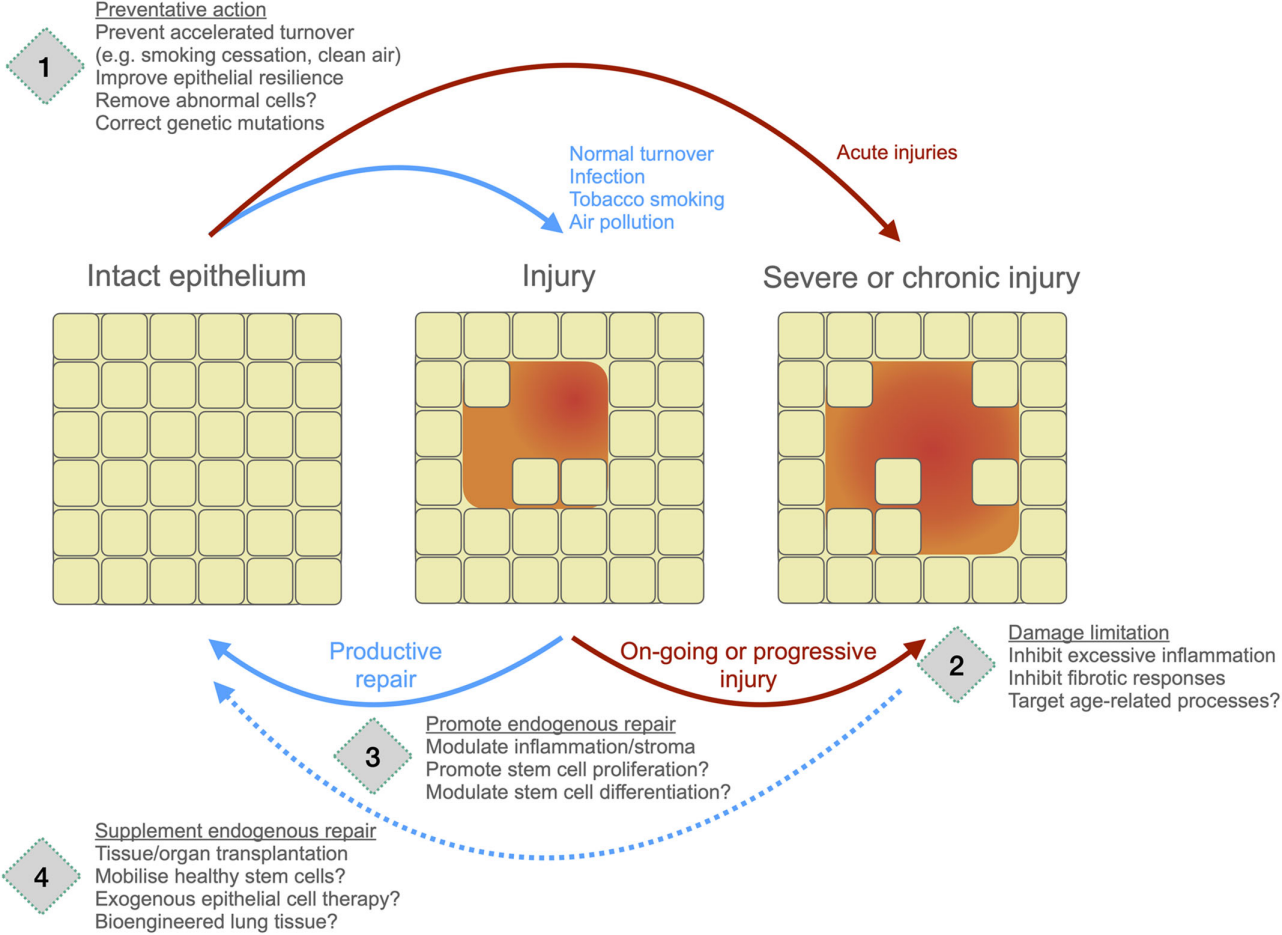
**Opportunities for the development of pro-regenerative therapies across the natural history of lung injury, repair and disease.** During homeostasis, lung epithelia have a baseline rate of tissue renewal, which is increased by exposure to environmental toxins. Repetitive injury reduces the probability of productive repair and increases risk of chronic lung disease. Future pro-regenerative therapies might (1) protect the epithelium from injury, (2) limit damage to make productive repair more likely, (3) promote endogenous repair processes, or (4) supplement repair through cell, tissue or organ transplantation.

To date, the regenerative medicine approach has seen limited clinical application in respiratory medicine. However, strategies are being developed to protect healthy lung tissue prior to the inception of disease, to promote the function of endogenous lung stem cells either directly or through host microenvironmental modification, and to initiate repair using exogenous autologous epithelial cell transplantation (Fig. 2). As well as impact in chronic lung diseases, the development of these pro-regenerative therapies also has the potential to improve outcomes for patients with acute airway or lung injuries.

Numerous issues will be vital for the advancement of pro-regenerative medicines in the lung context. First, emerging 3D models of human lung tissue can be used to better understand the considerable heterogeneity of regenerative responses between individual patients. Second, creating a microenvironment to foster regeneration within the lung might improve outcomes with existing or novel therapeutics. A better understanding of lung epithelial-immune interactions during development and resolving repair would be beneficial in this context, as would a more comprehensive understanding of the role of ageing in lung regeneration. Finally, successful pre-clinical lung epithelial cell expansion protocols will need modification in terms of GMP manufacture, scale and transplantation methods before clinical benefit. In the longer term, these data will also be informative for tissue- and organ-scale bioengineering applications.
